# *De Novo* Iron Oxide Hydroxide, Ferrihydrite Produced by* Comamonas testosteroni* Exhibiting Intrinsic Peroxidase-Like Activity and Their Analytical Applications

**DOI:** 10.1155/2019/7127869

**Published:** 2019-03-28

**Authors:** Amany Ahmed, Adam Abagana, Daizong Cui, Min Zhao

**Affiliations:** ^1^College of Life Science, Northeast Forestry University, Harbin 150040, China; ^2^Botany Department, Faculty of Science, Menoufia University, Shebin El-koom 32511, Egypt

## Abstract

Natural enzyme mimics have attracted considerable attention due to leakage of enzymes and their easy denaturation during their storage and immobilization procedure. Here in this study, for the first time, a new iron oxide hydroxide, ferrihydrite – Fe_1.44_O_0.32_ (OH) _3.68_ magnetic nanoparticles were synthesized by bacterial strain named* Comamonas testosteroni*. The characterization of the produced magnetic nanoparticles was confirmed by transmission electron microscopy (TEM), Fourier-transform spectroscopy (FTIR), X-ray diffraction (XRD), and magnetization hysteresis loops. Further, these extracted nanoparticles were proven to have biogenic magnetic behavior and to exhibit enhanced peroxidase-like activity. It is capable of catalyzing the oxidation of 3, 3′, 5, 5′-Tetramethylbenzidine (TMB) by H_2_O_2_ to produce blue color (typical color reactions). Catalysis was examined to follow Michaelis-Menton kinetics and the good affinity to both H_2_O_2_ and TMB. The *K*_m_ value of the Fe_1.44_O_0.32_ (OH) _3.68_ with H_2_O_2_ and TMB as the substrate was 0.0775 and 0.0155 mM, respectively, which were lower than that of the natural enzyme (HRP). Experiments of electron spin resonance (ESR) spectroscopy proved that the BMNPs could catalyze H_2_O_2_ to produce hydroxyl radicals. As a new peroxidase mimetic, the BMNPs were exhibited to offer a simple, sensitive, and selective colorimetric method for determination of H_2_O_2_ and glucose and efficiently catalyze the detection of glucose in real blood samples.

## 1. Introduction

Natural enzymes, which are biological catalysts, have noticeable advantages such as high specificity to the substrate and high efficiency under mild conditions. However, they have some severe disadvantages such as the easy denaturation by environmental changes as their catalytic activity based on their native protein; hence, they can be easily digested by proteases and also the high price and the time-consumption of their preparation and purification [1]. Therefore, a lot of efforts have been created to expand the natural enzymes to enzyme mimetic as an efficient approach to solve the problems of these enzymes for procedural applications [2–5].

There is growing evidence that nanoparticles can act as enzyme mimics as oxidase, peroxidase, or catalase mimetics which are distinguished by low cost, high tenability, and stability [6]. Also, they can be utilized in environmental chemistry, bioassays, and future applications [4, 7, 8]. There are many researches about chemical synthesis of inorganic nanomaterials exhibiting enzyme—like activity such as FeSe, CuO, CeO_2_, CoFe_2_O_4_, Co_3_O_4_ nanoparticles [9]. Thus, these mimic enzymes can replace natural enzymes used in many fields such as colorimetric detection, biosensor, immunoassays, and pollutant degradation [10].

Fe_3_O_4_ is considered the surprising discovery of nanoparticles because it has the intrinsic peroxidase-like activity identical to that of the natural enzyme, horseradish peroxidase (HRP) [7], so the inorganic nanomaterial's field as peroxidase mimics has attracted the researchers attention. For example, a study for indicating the ability of Fe_3_O_4_ MNPs [7], iron phosphates [11], and iron chalcogenides, Prussian blue (PB) [12] to catalyze the reactions of the typical peroxidase chromogens TMB, DAB and OPD in H_2_O_2_ presence has been accomplished. Most of these studies have used these nanoparticles as peroxidase mimetic for detection of H_2_O_2_ and glucose [11, 13, 14]. However, synthesis of these artificial nanoparticles is expensive and environmentally undesirable as it involves high temperature and produces so many toxic reagents [15] due to using organic solvents at high temperatures in the synthetic methods, such as sol-gel method, reverse miceller method, and the thermal decomposition of organometallic iron method.

Recently, microbial synthesis of MNPs has attracted great attention due to the avoidance of toxic substances production. Such synthesis is considered an ecofriendly process because it occurs in water under room temperature, pressure, and near to neutral pH [15].

Production of iron-containing nanoparticles by microorganisms is most advantageous because biogenic particles are more biocompatible and uniform in size than inorganic particles synthesized by chemical methods which made them preferable in medical areas. Moreover, processes of biosynthesis can be carried out under conditions of pressure and ambient temperature that are more simple than that of chemical syntheses and can display as an ecofriendly, cost-effective, and prospective way for more progress [16, 17]. Biological agents as microorganisms secrete large amounts of enzymes which reduce metals and can be responsible for nanoparticles synthesis [18–20].

Numerous studies have detected that many microorganisms as bacteria and fungi can form biogenic magnetic nanoparticles MNPs [15, 21]. Magnetotactic bacteria produce intracellular linear chains of nanosized magnetic organelles called magnetosomes [22]. Bacterial magnetosomes which are biomineralized in magnetotactic bacteria are natural inorganic ferromagnetic nanoparticles in the range of 35~120 nm within the single-domain size [23–25]. The presence of an organic membrane that contains magnetosome-specific proteins some of which are responsible for the biomineralization process [26, 27], enveloping the magnetite particles and the regular morphology, distinguishes the bacterial magnetite particles from artificially synthesized magnetite [28], in addition to ability of MTB to control the magnetic nanocrystal assemply through the magnetosome chains [29]. Each magnetosome consists of a magnetite (Fe_3_O_4_) or greigite (Fe_3_S_4_) crystal. It was reported that the magnetosomes play a role in iron storage and are considered as an eminent model to dissect the organization of the molecular landscape of bacterial organelle [30, 31] and in eliminating the toxicity of free ions in a cell leading to the production of toxic radicals because of Fenton reaction [32, 33]. Magnetosomes can also scavenge reactive oxygen species [34]. Based on the Fenton reaction, Fe^2+^ can catalyze the conservation of H_2_O_2_ which is considered a powerful and potential harmful oxidizing agent to OH radicals. Furthermore, these biogenic MNPs would be anticipated to show low cost, high chemical purity, uniform and fine particle size in addition to good biocompatibility without modification of the surface [35] which make them a promising magnetic nanomaterial in gene targeting and drug, biomolecule immobilization and wastewater treatment [36, 37]. We attempt to examine BMPs' intrinsic enzyme mimetic activity similar to that found in MNPs.

In the present paper, we have developed a facile, green method for the biogenic synthesis of iron oxide nanoparticles from the bacterial stain* Comamonas testosteroni*. The iron oxide hydroxide, ferrihydrite nanoparticles were evaluated for peroxidase activity by catalyzing the oxidation of peroxidase substrate 3, 3′, 5, 5′-tetramethylbenzidine (TMB) by hydrogen peroxide, producing blue color product, which can be detected by naked eye or spectroscopy. Further, a sensitive and selective colorimetric method for glucose detection was developed, based on the downregulating activity of glucose towards the peroxidase activity of iron oxide nanoparticles. The developed method provides sufficiently good sensitivity for screening and monitoring of glucose in real blood samples.

## 2. Materials and Methods

### 2.1. Chemicals

All chemicals were of analytical grade and used as received without further purification. 3, 3′, 5, 5′-Tetramethylbenzidine (TMB) was purchased from BIOSHARP company, glucose oxidase (GOx, from* Aspergillus niger*, ≥100 U/mg), and DMPO (5, 5-dimethyl-1-pyrroline N-oxide) were purchased from Sigma-Aldrich (St. Louis, USA). Glucose, fructose, lactose, maltose, cholesterol, inositol, ascorbic acid, vitamin B1, and vitamin B9 were purchased from Beijing Chemical Reagent Company (Beijing, China). H_2_O_2_ and other regeants were obtained from Boyue Biological Reagent Co. (Harbin, China).

### 2.2. Morphological and Analytical Characterization of the Magnetosomes

The bacterial cells of* C. testosteroni* were confirmed to contain intracellular magnetosome during the growth period in batch culture of 48 hours. The isolated bacterial strain of* C. testosteroni* was grown in a culture medium supplemented with 0.74 g sodium succinate, 0.25 g NaNO_3_, 0.12 g sodium acetate, 0.05 g sodium thioglycolate, 5 mL 0.01 M ferric citrate solution, 5.0 mL Wolfe's mineral solution, and 10 mL Wolfe's vitamin solution; pH of the culture medium was adjusted to 5.5-6.2 and autoclaved at 121°C for 20 min. Afterwards, the bottles were cooled, inoculated, and incubated at 30°C at static state for four days. After the incubation period, the bacterial cultures were centrifuged at 10000 rpm for 5 min and the pellets were washed three times with distilled water and then suspended in PBS buffer.

### 2.3. Preparation of the Nanoparticles Produced

The magnetosomes of the bacterial cells were extracted to evaluate their characteristics. The extraction was done by ultrasonication through an ultrasonic cracker (180 W, 20 s work, 15 s interval, 200 repetitions, NINGBO SCIENTZ BIOTECHNOLOGY CO.,LTD), this physical method was followed for cell dispersion and extraction of the magnetosomes. The magnetic particles, released from the bacterial cells, were harvested by permanent magnet. For characterization, the products of the output black solid were rinsed with water three times then separated and harvested by a strong permanent magnet. The debris of the cells and other impurities were removed with several washes with PBS buffer.

### 2.4. Characterization of the BMNPs Produced by the Bacterial Strain

The morphology and size of BMNPs were checked by transmission electron microscopy (TEM, FEI/Philips TCNAI G2) at an accelerating voltage of 200 kV and direct magnification of×250,000. The composition and phase of BMNPs were recognized by powder X-ray diffraction (XRD) on an D/max-rB X-ray diffractometer (Rigaku, Japan) using Cu K*α* radiation (*λ*=1.5418 Å). Fourier transform infrared (FT-IR) spectra of the nanoparticles were recorded in the range of 350-7800 cm^−1^ using FT-IR spectroscopy (Nicolette-6700). Room temperature-magnetic experiments were performed on a vibrating sample magnetometer (Model 3900, Princeton Measurements Corporation, sensitivity is 5.0×10−10 Am2). The hysteresis loop was measured between +500 and −500 mT with an average time of 400 ms. Saturation magnetization (Ms) and saturation remanence (Mrs) were determined after correction for paramagnetic phases.

### 2.5. Kinetic Analysis

Unless otherwise stated, steady state kinetic measurements were carried out in time-drive mode by monitoring the absorbance change at 652 nm on a Lambda 750 UV-Visible-near infrared (UV-Vis-NIR) spectrophotometer (PerkinElmer, USA). Experiments were carried out using 30 *μ*g/mL BMNPs in 3-mL reaction buffer solution (0.2 M acetic acid (HAc)-sodium acetate (NaAc) buffer (Fe_1.44_O_0.32_ (OH) _3.68_) NPs; PH 3.2, 50°C) in the presence of 40 mM L^−1^ TMB and 600 mM L^−1^ H_2_O_2_ as substrate. In order to display the kinetic characteristics, the velocity changes of the reaction used with changing concentrations of TMB and a fixed concentration of H_2_O_2_ or vice versa were obtained. The apparent kinetic parameters were calculated based on the Michaelis–Menten equation. The Michaelis–Menten constant was calculated using Lineweaver–Burk plots of the double reciprocal of the Michaelis–Menten equation, 1/v= (K_m_/V_max_) · (1/[S]) +1/V_max_, where V is the initial velocity, V_max_ is the maximum reaction velocity, [*S*] is the substrate concentration, and K_m_ is the Michaelis constant.

### 2.6. ESR Spectroscopy Measurements

The use of ESR technique was performed to indicate hydroxyl radicals (•OH) formed during the decomposition of H_2_O_2_ induced by BMNPs. Due to its diamagnetic property, 5,5-dimethyl1-pyrroline N-oxide (DMPO) is capable of trapping these short-lived •OH and readily forming stable spin adducts DMPO/•OH. BMNP sample was mixed with DMPO in the standard buffer of pH 3.2, and the reaction was triggered by addition of H_2_O_2_, then the sample mixtures were transferred into a glass capillary and put in the ESR cavity. The spectra subtraction between the sample mixtures with and without H_2_O_2_ solution was conducted to obtain ESR spectra signal of spin adducts DMPO/•OH. ESR measurements were carried out using Bruker EMX ESR spectrometer (Billerica, MA) at ambient temperature with 20-mW microwave power.

### 2.7. H_2_O_2_ and Glucose Detection

Typical colorimetric analysis for H_2_O_2_ detection was performed as follows: 60 *μ*L of 40 mM L^−1^ TMB, 100*μ*L of 0.3 mg mL^−1^ BMNPs, and 200 *μ*L of H_2_O_2_ with different concentrations added to 2,640 *μ*L of buffer (0.2 M acetate buffers, pH 3.2). Afterwards, the mixed reaction solution was detected using adsorption spectroscopy measurement. In a control experiment, 100 *μ*L of water was used instead of nanoparticles.

For the glucose detection performing as follows in two steps: (a) 20 *μ*L of 5.0 mg mL^−1^ GOx and 200 *μ*L of with different concentrations of glucose in 10 mM HAc-NaAc buffer (pH 5.5) were incubated at 37°C for 30 min; (b) 30 *μ*L of 1.0 mg mL^−1^ Fe_1.44_ O_032_(OH)_3.86_ NPs, 50 *μ*L of 40 mM L^−1^ TMB and 1000 *μ*L of 0.2 M HAc-NaAc buffer (pH 3.2) were added to the above 220 *μ*L glucose reaction solution; and (c) the mixture was incubated at 50°C for 30 min and then used for adsorption spectroscopy measurement at 652 nm. In the interference of glucose determination 5 mM L^−1^ sucrose, 5 mM L^−1^ lactose, 5 mM L^−1^ fructose, 5 mM L^−^cholestrol, 5 mM L^−^inositol, 5 mM L^−^ascorbic acid, 5 mM L^−1^ vitamin B1 and 5 mM L^−1^ vitamin B9 were independently used instead of 5 mM L^−1^ glucose in control experiments.

It is important to evaluate the proposed method specificity for detection of glucose in real samples. The specification experiments were performed using aforemention buffer. For determination of glucose in serum, the serum samples were firstly treated by centrifugation at 4000 rpm for 10 min. Afterwards, each sample was diluted two times using 10 mM L^−1^ PBS buffer (pH 7.2) for the subsequent work. The glucose in serum was measured according to the above procedure.

## 3. Result

### 3.1. Morphological and Analytical Characterization of Magnetosomes

**Figure 1:**
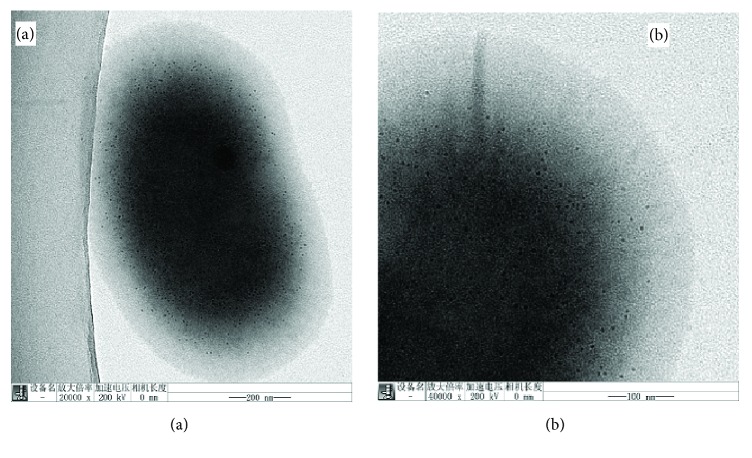
(a, b) TEM images of the bacterial cells of* C. testosterone.*

The bacterial strain of* C. testosteroni* was confirmed to contain intracellular magnetosome during the growth period in batch culture for 48 hours. TEM in Figure 1 showed that the bacterial cells are short rods in morphology with a mean width of 0.8 *μ*m and a mean length of 1.05 *μ*m. Also, too many magnetic nanoparticles present in each cell. The magnetic nanoparticles biosynthesis through the cultivation of* C. testosteroni* 48h is confirmed through TEM images.

### 3.2. Preparation and Characterization of the BMNPs Extracted from the Strain* C. testosteroni*

**Figure 2:**
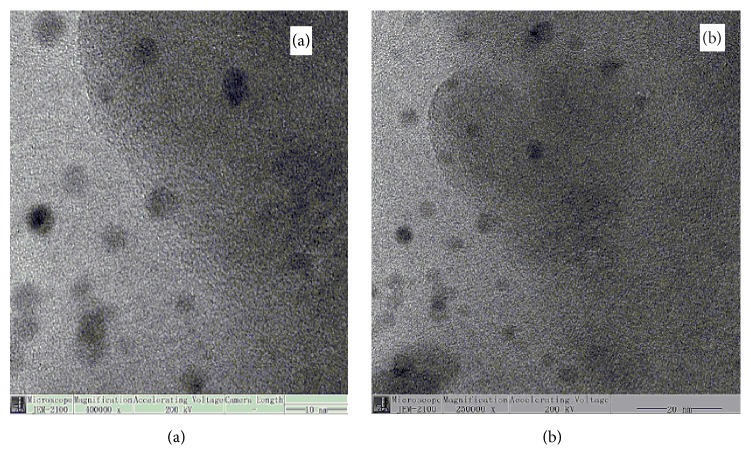
(a, b) TEM image of the BMNPs.

The magnetic nanoparticles in bacterial cells were extracted and purified by ultrasonication, ultracentrifugation, and magnet adsorption. TEM photographs of the Fe_1.44_ O_0_._32_ (OH) _3.86_ NPs have been given in Figure 2. The magnetite nanoparticles were intracellular because they were located inside the cytoplasmic membrane. The particles of the electron-dense in the cells of* C. testosteroni* were not arranged in a single chain; however, they were arranged at the center of the bacterial cytoplasm. The shape of nanoparticles was spherical as shown from the TEM and well dispersed in uniform size. Their shape is nearly hexagonal. It can be shown from the images of TEM that the particles have a very wide size distribution.

**Figure 3:**
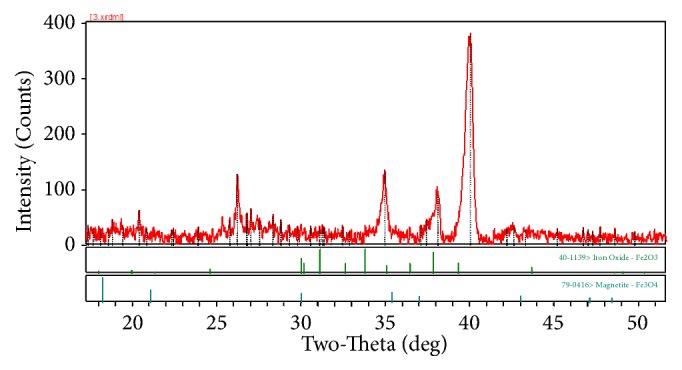
XRD patterns of Fe_1.44_O_0.32_ (OH) _3.86_ NPs.

The description of crystallographic structure and phase purity of the sample was examined by XRD measurement. XRD result showed that Fe_1.44_ O_032_(OH)_3.86_ was highly crystalline and all the diffraction peaks can be confirmed to be hexagonal crystalline phase of iron oxide hydroxide, ferrihydrite match well with the JCPDS card no. 01-073-8408 as shown in Figure 3. The pattern of XRD could be indexed in the pure phase of Fe_1.44_ O_0.32_ (OH) _3.86_. In the XRD pattern, the major diffraction peaks at 20.5°, 26°, 34°, 38°, and 40° could be indexed to the (261), (328), (290), (259), and (546) facet of the Fe_1.44_ O_032_(OH)_3.86_ phases.

**Figure 4:**
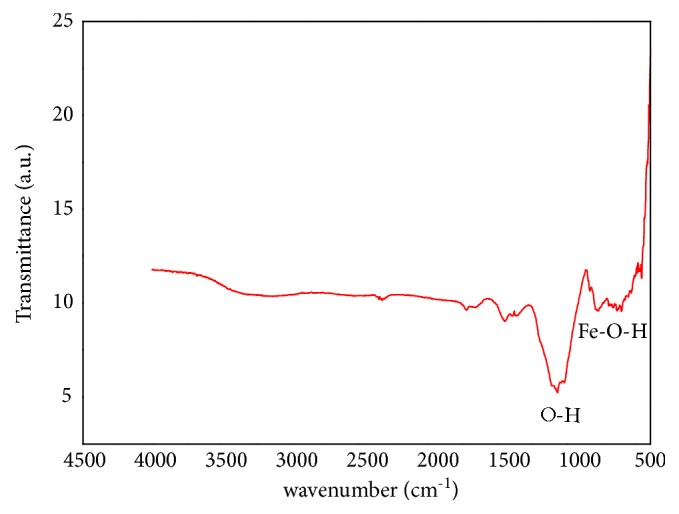
FT-IR spectra of the extracted magnetosomes from* C. testosteroni*.

Furthermore, the nanoparticles synthesized by C* testosteroni* were characterized by FT-IR spectroscopy. FT-IR spectroscopy is an important tool to know the functional group of any organic molecule. As shown in Figure 4 the absorption peaks at wavelength 1060, 1384, and1610 cm^−1^ were assigned to the O–H. The other absorption peaks at wavelength 671, 847 cm^−1^ were attributed to the characteristic Fe–O vibration. There is a typical band of *α*-FeOOH registered at 780 cm^−1^ which can be attributed to Fe–O–H bending vibrations.

**Figure 5:**
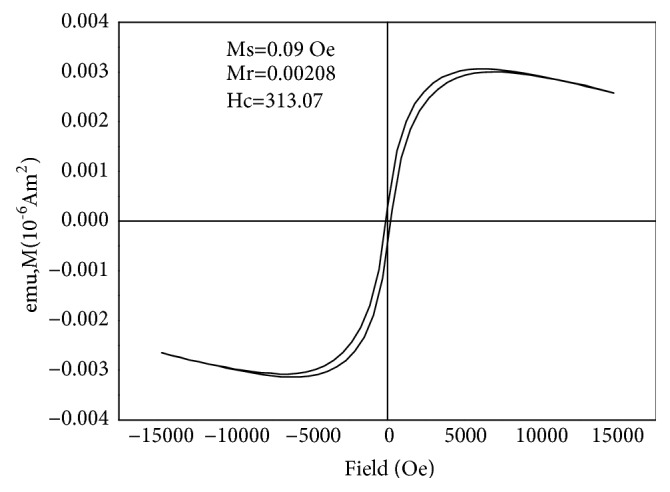
Room temperature hysteresis loop of BMNPs.

The results here indicate that there is well stabilization of the iron oxide nanoparticles. The magnetic properties of Fe_1.44_O_0.32_ (OH) _3.86_ were investigated with a vibrating sample magnetometer (VSM). The saturation magnetization (Ms) is 0.09 emu/g at room temperature and the magnetic coercivity Hc=313.07 Oe is soft magnetic material. The residual magnetic strength (Mr=0.00208 emu/g) is close to zero. The hysteresis loop of the BMNPs samples is protruded as shown in the Figure 5 and the values of hysteresis parameters such as the ratios of Mrs/Ms were deduced as 0.023. The significant softening of the magnetic property is caused depending on the nanoparticles small size. Fe_1.44_O_0.32_ (OH) _3.86_ NP has superparamagnetism and is easy to be separated from the solution under the external magnetic effect. Moreover, the saturation magnetization will be increased by decreasing the size of the particle [38].

### 3.3. The Peroxidase-Like Activity of the Fe_1.44_O_0.32_ (OH) _3.86_

**Figure 6:**
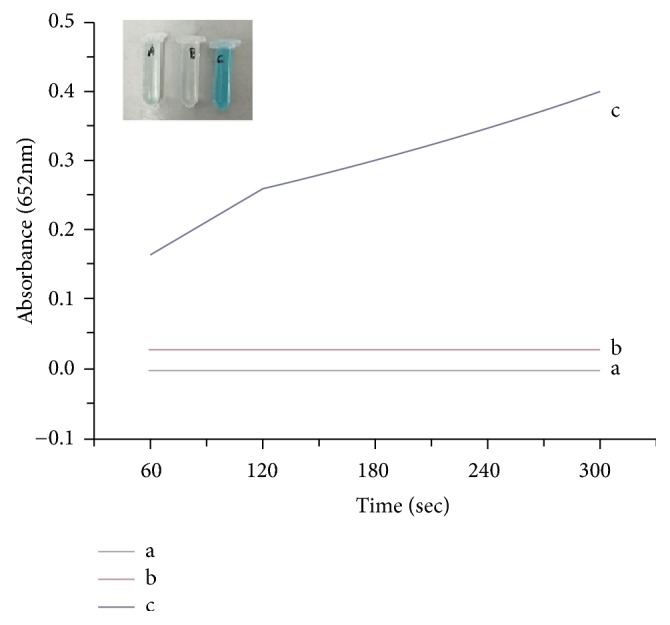
Time-dependent absorbance changes at 652 nm of TMB in different reaction systems: (a) TMB+H_2_O_2_, (b) TMB+BMNPs, and (c) TMB+BMNPs+H_2_O_2_. Reaction conditions were 40 mM L^−1^ TMB, 30 *μ*g mL −1 BMNPs, 200 mML^−1^ acetate buffers, and PH 3.2 at 50°C.

The peroxidase-like activity of the Fe_1.44_O_0.32_ (OH) _3.86_ was estimated by the catalytically oxidation of typical (TMB) by H_2_O_2_. When Fe_1.44_O_0.32_ (OH) _3.86_ was added to TMB in the presence of H_2_O_2_, a typical color could be seen as in (Figure 6). On the other hand, there were slight color variances in the absence of Fe_1.44_O_0.32_ (OH) _3.86_. Taking TMB as an example, a conspicuous ascension at 652 nm could be monitored. A^652^ nm in Fe_1.44_O_0.32_ (OH) _3.86_ –TMB–H_2_O_2_ system was at least 3-fold higher than that in the TMB–H_2_O_2_ and Fe_1.44_O_0.32_ (OH) _3.86_ –TMB systems, suggesting that Fe_1.44_O_0.32_ (OH) _3.86_ had significant peroxidase-like catalytic activity. Also, the absorbance at 652 nm increased with increasing H_2_O_2_ concentration (Figure 8(c)). Thus, the Fe_1.44_O_0.32_ (OH) _3.86_ could be potentially used as an effective enzyme mimic catalyst in applications of biochemical because H_2_O_2_ is the product of many enzymatic reactions of important biochemical substance, such as glucose.

**Figure 7:**
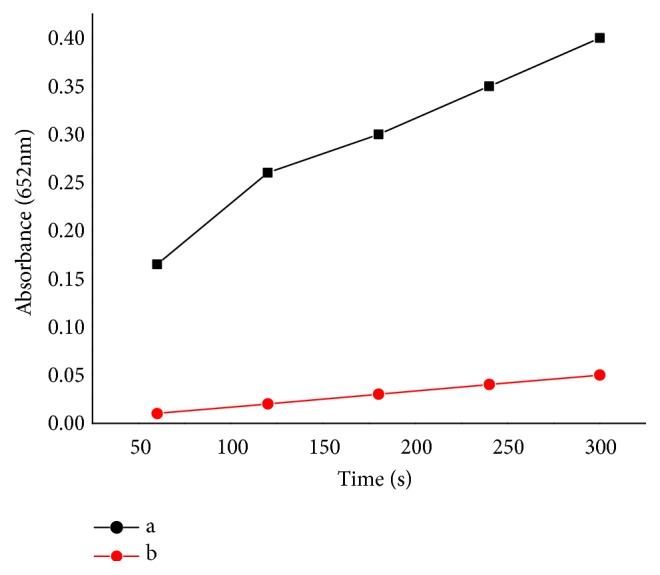
The time-dependent absorbance (at 652 nm) of (a) Fe_1.44_O_0.32_ (OH) _3.86_ NPs and (b) the leaching solution.

In order to indicate that the intrinsic peroxidase-like catalytic activity of the Fe_1.44_O_0.32_ (OH) _3.86_ NPs is due to the intact NPs rather than the free metal ions in leaching solution, the BMNPs were incubated in the reaction buffer for 10 min and then the NPs were removed from solution by centrifugation at 10000 r/min for 10 min to prepare a leaching solution. The supernatant of the aqueous solution of Fe_1.44_O_0.32_ (OH) _3.86_ NPs was tested towards the TMB oxidation reaction. The leaching solution had no activity (Figure 7), demonstrating that the intrinsic peroxidase-like activity cannot be attributed to leaching of iron ions into solution, but occurred on the surface of the NPs according to our experimental results. Our result showed that the leaching solution had no activity.

### 3.4. Effect of PH, Temperature, and H_2_O_2_ Concentration on BMNPs Activity

**Figure 8:**
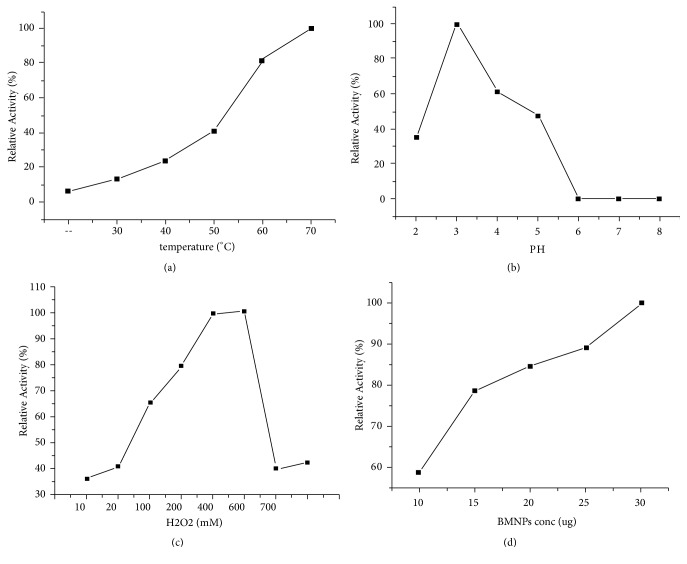
Dependency of peroxidase-like activity on (a) temperature effect, (b) PH effect, (c) H_2_O_2_ concentration, and (d) BMNPs concentration. For (d) the oxidation activity rate of BMNPs was determined in 200 mM L^−1^ acetate buffer (pH 3.2) with a series of BMNP concentrations that are 10, 15, 20, 25, 30 *μ*g mL^−1^, respectively. TMB (25 mM L^−1^) and H_2_O_2_ (100 mM L^−1^) were added to initiate the reaction. (a)(b)-(c)-(d) Experiments were carried out using 30 *μ*g mL ^−1^ BMNPs in the reaction buffer above with 25 mM L^−1^ TMB. The H_2_O_2_ concentration was 100 mM L^−1^ at 30°C unless otherwise stated. The maximum point in each curve was set as 100%.

The peroxidase-like activity of BMNPs was estimated with changing the pH from 1 to 8, the temperature from 25 to 70°C, and H_2_O_2_ concentration from 0.01 M to 1 M. It has been shown in Figure 8 that the maximum catalytic activity of the Fe_1.44_O_0.32_ (OH) _3.86_ was obtained under the following optimal conditions: pH 3.2, 50°C, 600 mM L^−1^ and 30 mg L^−1^Fe_1.44_O_0.32_ (OH) _3.86_. The reaction time was set as 5 min.

### 3.5. Kinetic Analysis of BMNPs Activity

**Figure 9:**
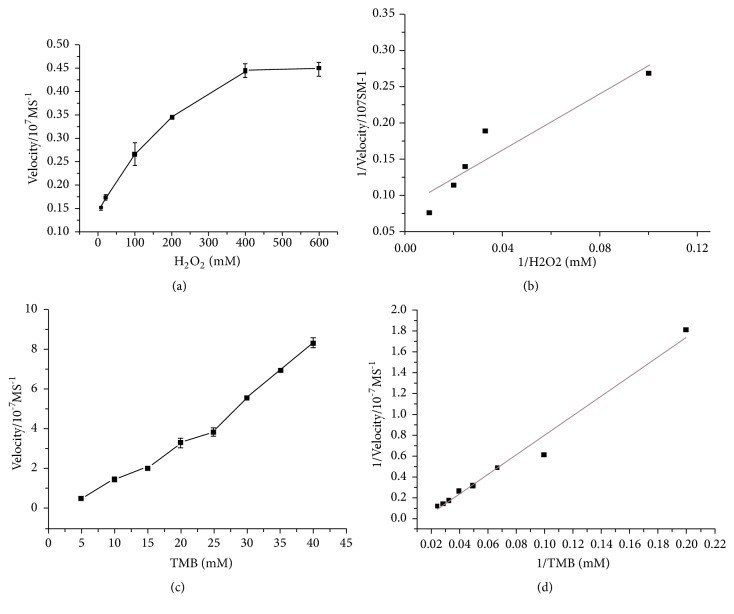
Steady state kinetic assay of BMNPs. (a) The concentration of TMB was 40 mM L^−1^ and the H_2_O_2_ concentration was varied. (c) The concentration of H_2_O_2_ was 600 mM L^−1^ and the TMB concentration was varied. (b, d) Double reciprocal plots of activity of BMNPs at a fixed concentration of one substrate against differing concentration of the other substrate for TMB and H_2_O_2_.

Typical Michaelis–Menten curves (Figures 9(a)–9(d)) were obtained in a certain range of TMB or H_2_O_2_ concentrations. With the Line weaver-Burk equation, the important enzyme kinetic parameters such as Michaelis–Menten constant (K_m_) and Maximum initial velocity (V_max_) were obtained in Table 1. K_m_ value is a binding affinity parameter describing the catalyst and the affinity of catalyst to analyzed substrate, that means the lower the K_m_ value the stronger the affinity of catalyst to substrate. K_m_ was recognized as an indicator of enzyme affinity to substrates. The apparent K_m_ value of Fe_1.44_O_0.32_ (OH) _3.86_ NPs with H_2_O_2_ as the substrates was 26 times lower than that for HRP.

The peroxidase-like catalytic activity of BMNPs was investigated using the typical Michaelis– Menten curves, and steady state kinetics are shown in Figures 9(a) and 9(b). The kinetic data were obtained by changing the concentration of one substrate while keeping the concentration of the other substrate constant. A series of initial reaction rates were counted and applied to the double reciprocal of the Michaelis–Menten equation, 1/v=(K_m_/ V_max_)·(1/[S])+1/V_max_, where v is the initial velocity, [S] is the concentration of the substrate, Km is the Michaelis–Menten constant, and Vmax is the maximal reaction velocity. The K_m_ and V_max_ were obtained using Lineweaver–Burk plots. To further investigate the mechanism of BMNPs catalysis, their activity over a range of TMB and H_2_O_2_ concentrations was measured. The double reciprocal plots of the initial velocity versus concentration of one substrate were gained over concentration range of the other substrate (Figures 9(c) and 9(d)).

### 3.6. Study of Free Radical Formation by ESR

**Figure 10:**
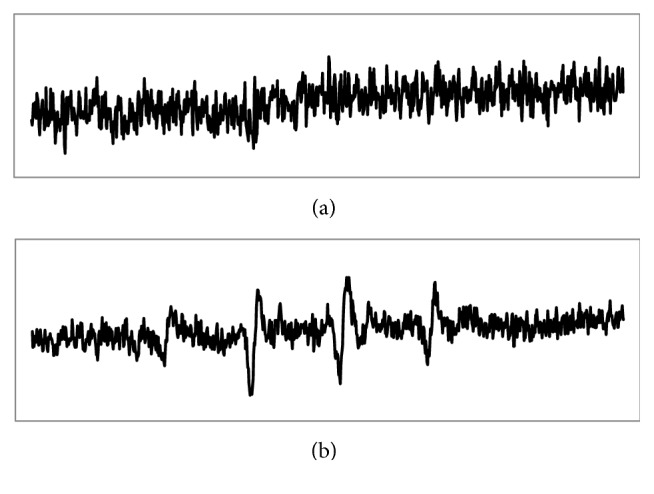
ESR spin-trapping spectra of H_2_O_2_- Fe_1.44_O_0.32_ (OH) _3.86_ system in the (a) absence and (b) presence of TMB. Conditions: 100 mM L^−1^ H_2_O_2_, 400 mML^−1^ DMPO, 25 mM L^−1^ TMB, 30 mg L^−1^ and 0.2 M NaAc buffer.

To confirm the generation of ^*∙*^OH radicals from H_2_O_2_ decomposition an ESR experiment was employed. According to the result here there is evidence that H_2_O_2_ alone did not produce OH radical. Although after addition of BMNPs helped the production of OH radicals from H_2_O_2_. As shown in Figure 10, the ESR spectra in the Fe_1.44_O_0.32_ (OH) _3.86_ –H_2_O_2_ system displayed a typical fourfold characteristic peak of the DMPO–^*∙*^OH adducts with an intensity ratio of 1 : 2 : 2 : 1. However, the DMPO–^*∙*^OH adducts signal intensity in the control experiment of the BMNPs absence.

### 3.7. Detection of H_2_O_2_ and Glucose

**Figure 11:**
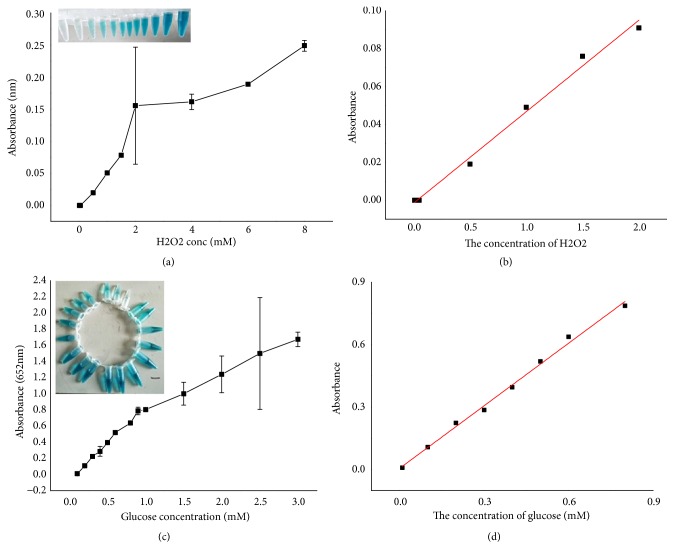
(a, b) Calibration plots of the absorbance versus the concentrations of H_2_O_2_ (a) and glucose (b) under the optimum conditions. Inset: the corresponding linear calibration plots for H_2_O_2_ (a) and glucose (b) detection.

A simple and sensitive colorimetric method was developed to detect H_2_O_2_ and glucose, depending on the peroxidase-like activity of Fe_1.44_O_0.32_ (OH) _3.86_NPs, and was applied to the glucose detection in human serum. As shown in Figures 11(a) and 11(b) the absorbance of TMB oxidation intermediates at 652 nm was good linear relationship with the concentration of H_2_O_2_ in the range of 1×10^−3^–2.5 mM. The linear regression equation was A = 0.4832 [H_2_O_2_] + 0.00158, and the correlation coefficient R was 0.98977. The detection limit of this assay for H_2_O_2_ was 2.19 *μ*M. H_2_O_2_ could be generated by the reaction that glucose oxidase (GOx) could catalyze the glucose oxidation in the presence of O_2_; that is, when coupled with the glucose catalytic reaction by GOx, the above TMB- H_2_O_2_ catalytic reaction could be used to indirectly detect glucose by aid of Fe_1.44_O_0.32_ (OH) _3.86_ NPs as the peroxide-like enzyme. As shown in Figure 11(c), with the increase of the concentration of glucose in the range of 0–1.2 mM, the absorbance at 652 nm increased gradually.  Figure 11(d) displays the standard curve of glucose. The linear regression equation was A = 1.00102 [glucose] + 0.00638 with a correlation coefficient of 0.9944 and the linear range for glucose from 1×10^−3^–1.2 mM. The detection limit was as low as 2.618*μ*M.

**Figure 12:**
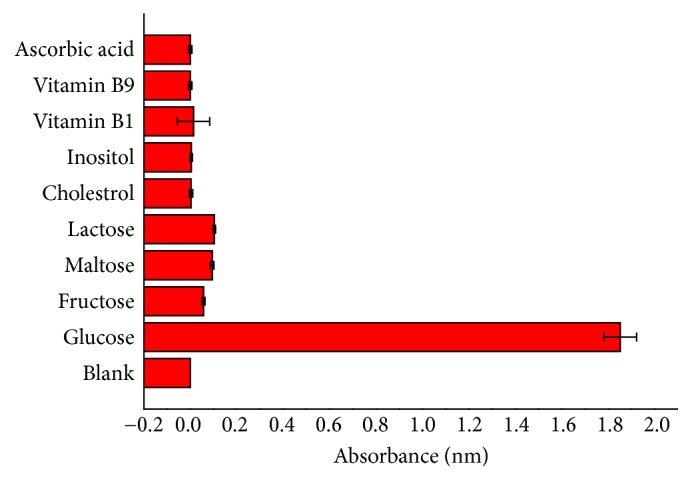
Specificity analysis of the colorimetric method for glucose detection.

Furthermore, the specificity of the glucose detection assay was investigated by conducting control experiments using 5 mM L^−1^ maltose, 5 mM L^−1^ D-fructose, 5 mM L^−1^ lactose, 5 mM L^−1^ cholestrol, 5 mM L^−1^ inositol, 5 mM L^−1^ ascorbic acid, 5 mM L^−1^ vitamin B1, and 5 mM L^−1^ vitamin B9 instead of 5 mM L^−1^ glucose. However, the concentrations of the substances were as fold as that of glucose; the response of these interfering substances was negligible compared with that of glucose (Figure 12). Due to these results, the colorimetric method employed here had high specificity for glucose.

### 3.8. Glucose Detection in Human Serum

In view of the high specificity and high sensitivity towards glucose, the developed method was used to determine glucose concentration in human serum. Four samples of the serum with different glucose concentrations were diluted two times to make the glucose concentration in serum samples in the linear regression equation of glucose range. As shown in Table 3, the experimental values were in agreement with those provided by the hospital. The accuracy of the method was assessed by the spiked recovery test.

## 4. Discussion

Because of the dual functionality of the magnetic nanoparticles (MNPs) as peroxidase mimetic and magnetic separation agents, there is particular interest of these particles [7]. Lately, biosynthesis of MNPs has become a useful method instead of traditional chemical procedures that utilize high temperatures, hazardous organic solvents and pressure; hence, they hurt the environment badly. Instead of this, the development of green synthesis and ecofriendly approaches to biosynthesize MNPs using microorganisms has obtained considerable attention. Some microorganisms were confirmed to manufacture ferromagnetic nanoparticles with uniform particle size and single domain such as magnetotactic bacteria [36, 39, 40] and nonmagnetotactic bacteria [37, 41]. The biogenic MNPs manifested superior performances compared with artificial magnetic nanoparticles (AMNPs) [35].

In this study, we have isolated MNP-producing bacterial strain recognized as* Comammonas testosteroni*; new MNPs were extracted from the strain that were characterized to be hexagonal in shape, 80 nm in size, affirmed after 48 h, with the chemical structure Fe_1.44_O_0.32_ (OH) _3.86_ and ferromagnetic behavior.

Moreover, we provide the first report that Fe_1.44_O_0.32_ (OH) _3.86_ MNPs possess intrinsic peroxidase-like activity comparable to that of an enzyme catalyzed reaction by demonstrating that (1) Fe_1.44_O_0.32_ (OH) _3.86_ MNPs catalyzed the reaction of peroxidase substrate such as TMB to give the same color changes as HRP; (2) the peroxidase-like activity of Fe_1.44_O_0.32_ (OH) _3.86_ MNPs was also H_2_O_2_, pH, and temperature dependent; (3) catalysis by Fe_1.44_O_0.32_ (OH) _3.86_ MNPs showed typical Michaelis–Menten kinetics; and (4) catalysis by Fe_1.44_O_0.32_ (OH) _3.86_ MNPs was in agreement with a ping-pong mechanism.

According to TEM in Figure 2, it has been shown that the particles of the electron-dense in the cells of* C. testosteroni* were hexagonal in shape but not arranged in a single chain; however, they were arranged at the center of the bacterial cytoplasm. Such results looked alike synthesized AgNPs inside the periplasmic space of the bacterial strains* Psuedomonas stutzeri* AG259 and* B. licheniformi* [42].

Considering the patterns of XRD of the BMNPs from C.* testosteroni*, the characteristic diffraction peaks within the range of 15 < 2*θ* < 65 can be indexed as pure Fe_1.44_O_0.32_ (OH) _3.86_ (JCPDS card no. 01-073-8408), and the sharp peaks illustrated that the product is well crystalline (Figure 3).

The FT-IR analysis was carried out to know the functional groups of the BMNPs as shown in Figure 4, the absorption peaks at wavelength 1060, 1384, and 1610 cm^−1^ were assigned to the O–H vibrations of absorbed H_2_O molecules or structural OH groups [43, 44]. The other absorption peaks at wavelength 671,847 cm^−1^ were attributed to the characteristic Fe–O vibration modes in *β*-FeOOH [43, 45, 46]. There is a typical band of *α*-FeOOH recorded at 780 cm^−1^ can be attributed to Fe–O–H bending vibrations in *α*-FeOOH[47].

The hysteresis curve of the BMNPs in Figure 5 was recorded at room temperature with a vibrating sample magnetometer. The main scientific parameters to describe the magnetism of ferromagnetic materials are the remnant magnetization Mr, the saturation magnetization Ms, and the coercivity Hc. The high H_c_ value can be evident for existing strong magnetic interactions between nanoparticles [45, 46]. The loop squareness ratio (Mr/Ms) was very low (< 0.5) indicating the magnetostatical interaction of the particles [48, 49] and also the presence of significant amount of SP particles [38]. Moreover, the saturation magnetization will be increased by decreasing the size of the particle [38].

In comparison with those exhibited by horseradish peroxidase (HRP), artificial magnetic nanoparticles (AMNPs) have been demonstrated to have peroxidase-like activity [7] and the synthesis of the MNP-magnetosome in the bacterial cell protecting it from H_2_O_2_ toxicity was also mentioned by [30]. In addition, the bacterial magnetosomes in* Magnetospirillum gryphiswaldense* MSR-1 exhibiting peroxidase-like activity to decrease the levels of intracellular reactive oxygen species (ROS) was also demonstrated by [34]. It is known that peroxidase can catalyze the peroxidase substrates oxidation to produce a typical color [30, 50]. To investigate the peroxidase-like activity of the extracted BMNPs from YN01, similar experiments were carried out. For the first time, we confirmed that the BMNPs extracted from the strain of C.* testosteroni* can be evaluated as peroxidase mimetic. Similar to the horseradish peroxidase (HRP) as a natural enzyme, the peroxidase-like catalytic activity of the Fe_1.44_O_0.32_ (OH) _3.86_ depends on temperature, pH and H_2_O_2_ concentration [7]. Previously, it has been reported that the catalytic oxidation of TMB with H_2_O_2_ in acidic solutions using Fe_3_O_4_ MNPs was much faster than in neutral and alkaline solutions [51]. Our current result indicated that the catalytic behavior of the Fe_1.44_O_0.32_ (OH) _3.86_ was dependent on the pH (Figure 8(b)). The catalytic activity of Fe_1.44_O_0.32_ (OH) _3.86_ increased with the increase of pH of Fe_1.44_O_0.32_ (OH) _3.86_ solution from 2.0 to 4.0. However, it was minimized with increase of pH from 5.0 to 6.0. Thus, 3.2 was chosen as the optimum pH value of Fe_1.44_O_0.32_ (OH) _3.86_ solution for the subsequent investigations. According to [51], the reason for increasing the enzyme activity is due to the Fenton's reagent (i.e., Fe^2+^/Fe^3+^ ions in solution) that can help the H_2_O_2_ breakdown as shown with the iron ions included in the Fe_3_O_4_ MNPs which might leak into the buffer of the reaction solution. Other causes may be attributed to the more stability of H_2_O_2_ in pH 3 ~ 4.5, but not in alkaline solution [52], and can be analyzed immediately to produce H_2_O and O_2_ as shown in

(1) $$\begin{array}{c} 2H_{2}O_{2}\to 2H2O+O2 \end{array}$$

Briefly, both the strong acid or alkaline environment deviating from the neutral environment for bacterial growth (pH = 6.75) may reduce the enzyme activity of BMPs [53]. As for the effect of temperature batch experiments were conducted from 20 to 70°C. As can be illustrated, the maximum catalytic activity of Fe_1.44_O_0.32_ (OH) _3.86_ was obtained at 50°C (Figure 8(a)). Here, the results showed that the signals increased as the reaction temperature increased and that in consistent with the result of [51]. Nevertheless, our study is different from some previous reports [53, 54] because as the temperature increased, the catalytic reaction increased. We also investigated the effect of H_2_O_2_ concentration on the catalytic behavior of the BMPs, the concentration of H_2_O_2_ was carried out from 0.01 M to 1 M. It has been found that the best concentration of H_2_O_2_ is 600 mM which is about six orders of magnitude higher than HRP to reach the maximal level of peroxidase activity; this demonstrated that the catalytic activity of BMNPs is more stable at high H_2_O_2_ concentration than that of HRP. If the concentration was greater than the optimum value, H_2_O_2_ showed some inhibitory effect. If the concentration was too low, H_2_O_2_ could not lead to follow-up of the enzymatic reaction [53]. Finally, the effect of the Fe_1.44_O_0.32_ (OH) _3.86_ concentration was investigated over the range of 10-30 *µ*g mL^−1^ as shown in (Figure 8(d)). It was found that the catalytic activity of Fe_1.44_O_0.32_ (OH) _3.86_ increased with increasing Fe_1.44_O_0.32_ (OH) _3.86_ concentrations in the range of 10 to 30 *µ*g mL^−1^. Finally, 30 *µ*g mL^−1^ of the Fe_1.44_O_0.32_ (OH) _3.86_ was chosen for subsequent experiments.

The apparent K_m_ values of Fe_1.44_O_0.32_(OH)_3.86_ NPs with H_2_O_2_ as the substrates was 26 times lower than that for HRP [7, 50], showing that Fe_1.44_O_0.32(_OH)_3.86_ NPs had higher affinity to H_2_O_2_ than HRP and the other reported nanomaterial with peroxidase-like activities. This is consistent with the observation that a lower concentration of H_2_O_2_ was needed to illustrate maximal activity for the Fe_1.44_O_0.32_(OH)_3.86_ NPs. Moreover, the K_m_ value of Fe_1.44_O_0.32_ (OH) _3.86_ NPs with TMB was also lower than that of HRP, indicating that Fe_1.44_O_0.32_ (OH) _3.86_ NPs had a higher binding affinity to TMB. This may be because the surface of Fe_1.44_O_0.32_ (OH) _3.86_ NPs has multiple active sites, while one HRP molecule has only one iron ion in the active site [7, 50]. Furthermore, the double reciprocal plots (Figures 9(b) and 9(d)) indicated the characteristic parallel lines of a ping-pong mechanism and implied that like HRP, the Fe_1.44_O_0.32_(OH)_3.86_ NPs bind and react with first substrate, then release first product before reacting with second substrate [7].

Iron and other free metal ions are significant cofactors for the enzymes of antioxidant defense as catalase, peroxidase, and SOD [54–56]. In order to test if the peroxidase-like activity of BMNPs is according to the intact nanoparticles but not the free metal ions leaching into solution, first BMNPs were incubated in the reaction buffer for 10 min and then separated from the solution with a strong magnet to prepare a leaching solution. Our results illustrated that the demonstrated peroxidase-like activity is produced from the surface properties of the nanostructure but not from the ion-leaching process.

Last reports have indicated that ^*∙*^OH radicals were obtained from the decomposition of H_2_O_2_ during the catalytic reaction of different nanozymes [57]. To confirm the generation of ^*∙*^OH radicals from H_2_O_2_ decomposition an ESR experiment was employed. It has been proved that iron oxide NPs transmit electron between pairs of various oxidation states of Fe^2+^/Fe^3+^ to drive their catalytic activity [58, 59]. In the present system, H_2_O_2_ molecules can be adsorbed on the surface of BMNPs and then activated by bound Fe2+ and Fe3+ to produce the •OH radical. The produced •OH radical might be stabilized by BMNPs through partial electron exchange interaction that may give the catalytic ability of BMNPs.

A simple and sensitive colorimetric method was developed to detect H_2_O_2_ and glucose, depending on the peroxidase-like activity of Fe_1.44_O_0.32_ (OH) _3.86_NPs, and was applied to the glucose detection in human serum. The linear regression equation was A = 0.4832 [H_2_O_2_] + 0.00158, and the correlation coefficient R was 0.98977. The detection limit for H_2_O_2_ was 2.19 *μ*M, which was much lower than other reported nanomaterials before such as Magnetite, Cobalt ferrite, and Nickel ferrite [60], Co_3_O_4_ nanowalls (GC) [61], and Fe_3_O_4_/chitosan (GC rotating electrode) [62].

H_2_O_2_ could be generated by the reaction that glucose oxidase (GOx) could catalyze the glucose oxidation in the presence of O_2_ that is mean, when coupled with the glucose catalytic reaction by GOx; the above TMB- H_2_O_2_ catalytic reaction could be used to indirectly expose glucose by support of Fe_1.44_O_0.32_ (OH) _3.86_ NPs as the peroxide-like enzyme. The linear regression equation was A = 1.00102 [glucose] + 0.00638 with a correlation coefficient of 0.9944, and the linear range for glucose from 1×10^−3^–1.2 mM. The detection limit was as low as 2.618 *μ*M which was even lower than that provided by the synthesized one CuS, H2TCPP–CdS and Au NPs as shown in (Table 2). The color variation was observed clearly by the naked eye (inset of Figure 11(c)). The Fe_1.44_O_0.32_ (OH) _3.86_ NPs-based detection system indicated a wide linear range and extremely high sensitivity to glucose.

To evaluate the diagnostic capability of the selected Fe_1.44_O_0.32_(OH)_3.86_NPs, we used real blood samples of human which had representative levels of glucose according to the normal, boundary, and high stage of hyperglycemia (normal; ≤ 5.6 mM, boundary; 5.6 ~ 7 mM, and high; >7 mM) [64]. The glucose concentration in serum samples of the healthy and diabetic persons is about 3–8 mM and 9–40 mM, respectively [65]. Therefore, the proposed colorimetric method by aid of Fe_1.44_O_0.32(_OH) _3.86_ NPs was able to detect the glucose concentration in human serum with high sensitivity, selectivity, and accuracy. As shown in Table 3, the values of the experiment were in agreement with those provided by the hospital. The accuracy of the method was assessed by the spiked recovery test.

## 5. Conclusion

In summary, we have synthesized for the first time novel magnetic nanoparticles namely iron oxide hydroxide, ferrihydrite designed as Fe_1_._44_O_0.32_ (OH) _3.86_ from the bacterial strain* Comamonas* genus using microbial synthesis method to avoid the production of toxic substances commonly produced by chemical synthesis methods and further characterized the extracted BMNPs and showed that it has magnetic behavior afterwards, proving that it possesses high peroxidase-like catalytic activity. The peroxidase-like activity of Fe_1_._44_O_0.32_ (OH) _3.86_ is dependent on temperature, pH. Moreover, we also detect that the BMNPs could be used for colorimetric detection of H_2_O_2_ and glucose, with high efficiency in real human serum glucose detection. This work will introduce new information of biogenic magnetic nanoparticles as peroxidase mimetic and facilitate their utilization in catalytic elimination and bioassays of biomedical applications.

## Figures and Tables

**Table 1:** Comparison of K_m_ and V_max_ between Fe_1.44_O_0.32_(OH)_3.86_ and HRP for H_2_O_2_ and TMB.

Catalyst	Substance	K_m_ (mM )	V_max_ (M·s−1)	Reference
Fe_1.44_O_0.32_(OH)_3.86_	H_2_O_2_	0.0775	0.197	This work
Fe_1.44_O_0.32_(OH)_3.86_	TMB	0.0155	6.88	This work
HRP	H_2_O_2_	3.70	8.71 × 10^−8^	[7]
HRP	TMB	0.434	1 × 10^−7^	[7]
Fe_3_O_4_ NP	TMB	0.099	0.66×10^−7^	[4]
Fe_3_O_4_ NP	H_2_O_2_	50	5×10^−7^	[4]

**Table 2:** Comparison of this work with other nanomaterial based-peroxidase mimics for the detection of glucose.

Nanomaterial	The linear range(*μ*M)	The detection limit(*μ*M)	References
CuS NPs	2–1800	120	[63]
H_2_TCPP–CdS NCs	18.75–100	7.02	[64]
Au NPs	18–1100	4	[65]
Prophyrin-ZnS	50-500	36	[66]
Fe_1.44_O_0.32_(OH)_3.86_	0.8-1200	2.618	This Work

**Table 3:** The determination of glucose content in human serum sample.

Samples	Provided by hospital (mM)	Experimental result (mM ± SD, n = 3)	Recovery (%)
1	6.3	6.45±0.035355	102.38%
2	5.7	5.4±0.212132	95%
3	6.4	5.7±0.141421	89%
4	8.0	8.52±0.296985	106.5%

## Data Availability

No data were used to support this study.
